# A Sequence Variation in *GmBADH2* Enhances Soybean Aroma and Is a Functional Marker for Improving Soybean Flavor

**DOI:** 10.3390/ijms23084116

**Published:** 2022-04-08

**Authors:** Linlin Qian, Hangxia Jin, Qinghua Yang, Longming Zhu, Xiaomin Yu, Xujun Fu, Man Zhao, Fengjie Yuan

**Affiliations:** 1 Hangzhou Sub-Center of National Soybean Improvement, Institute of Crop and Nuclear Technology Utilization, Zhejiang Academy of Agricultural Sciences, Hangzhou 310021, China; qianlinlin2022@163.com (L.Q.); jinhangxia@126.com (H.J.); tsingyang2009@163.com (Q.Y.); zlmsllzly@163.com (L.Z.); yuxm@zaas.ac.cn (X.Y.); fuxj@zaas.ac.cn (X.F.); 2 Key Laboratory of Information Traceability for Agricultural Products, Ministry of Agriculture and Rural Affairs of China, Hangzhou 310021, China; 3 The National and Local Joint Engineering Research Center for Bio-Manufacturing of Chiral Chemicals, College of Biotechnology and Bioengineering, Zhejiang University of Technology, Hangzhou 310014, China; mzhao@zjut.edu.cn; 4 Zhejiang Key Laboratory of Digital Dry Land Crops, Zhejiang Academy of Agricultural Sciences, Hangzhou 310021, China

**Keywords:** aromatic vegetable soybean, gene sequence comparison, HRM molecular marker, soybean breeding, CRISPR/Cas9 gene editing

## Abstract

The vegetable soybean (*Glycine max* L. Merr.) plant is commonly consumed in Southeast Asian countries because of its nutritional value and desirable taste. A “pandan-like” aroma is an important value-added quality trait that is rarely found in commercial vegetable soybean varieties. In this study, three novel aromatic soybean cultivars with a fragrant volatile compound were isolated. We confirmed that the aroma of these cultivars is due to the potent volatile compound 2-acetyl-1-pyrroline (2AP) that was previously identified in soybean. A sequence comparison of *GmBADH1/2* (encoding an aminoaldehyde dehydrogenase) between aromatic and non-aromatic soybean varieties revealed a mutation with 10 SNPs and an 11-nucleotide deletion in exon 1 of *GmBADH2* in Quxian No. 1 and Xiangdou. Additionally, a 2-bp deletion was detected in exon 10 of *GmBADH2* in ZK1754. The mutations resulted in a frame shift and the introduction of premature stop codons. Moreover, genetic analyses indicated that the aromatic trait in these three varieties was inherited according to a single recessive gene model. These results suggested that a mutated *GmBADH2* may be responsible for the aroma of these three aromatic soybean cultivars. The expression and function of *GmBADH2* in aromatic soybean seeds were confirmed by qRT-PCR and CRISPR/Cas9. A functional marker developed on the basis of the mutated *GmBADH2* sequence in Quxian No. 1 and Xiangdou was validated in an F_2_ population. A perfect association between the marker genotypes and aroma phenotypes implied that *GmBADH2* is a major aroma-conferring gene. The results of this study are potentially useful for an in-depth analysis of the molecular basis of 2-AP formation in soybean and the marker-assisted breeding of aromatic vegetable soybean cultivars.

## 1. Introduction

Vegetable soybean, which is also known as edamame in Japanese and maodou in Chinese, is popular among consumers because of its high nutritional value (e.g., a rich source of proteins, vitamins, and flavonoids), especially in East and Southeast Asian countries, including China, Japan, and Vietnam [1]. In the last decade, the vegetable soybean has become increasingly popular in the United States of America. Flavor is one of the most important quality traits affecting the economic value of vegetable soybean. In Japan, aromatic soybean is sold at a much higher market price than common soybean varieties because of its unique aroma [2]. 

More specifically, aromatic soybean has a potent popcorn-like or pandan-like aroma because it contains 2-acetyl-1-pyrroline (2AP), which is a volatile compound. Additionally, 2AP is the key flavor-related compound in diverse cereal products and vegetable-derived products. It is biosynthesized in various crops, including certain varieties of rice and soybean. In aromatic vegetable soybean, the seed 2AP content is reportedly 0.28–1.16 μg/g, with peak levels during the R6 and R7 stages, which is followed by a gradual decrease [3]. In fragrant rice grains, the 2AP content ranges from 0.03 to 0.55 μg/g [4]. The 2AP content in Sona mung bean increases along with the progression of growth stages, peaking at 0.19 ± 0.01 μg/g in seeds [5]. A previous study revealed that the 2AP content in aromatic winter melon WG-PB-259 is 25.90 ± 2.48 μg/g [6].

Several published studies suggested the aroma of various crops is most commonly controlled by a single recessive gene [7,8,9,10,11]. In the last decade, the genes responsible for aroma have been identified in several crops, including rice (*Oryza sativa*) [8,12], soybean (*Glycine max*) [7,9], sorghum (*Sorghum bicolor*) [11], cucumber (*Cucumissativus*) [10], winter melon, and coconut [13]. In these plant species, the major candidate gene was named *BADH2*, which encodes a betaine aldehyde dehydrogenase. Genetic studies on rice detected mutations in the betaine aldehyde dehydrogenase gene *OsBADH2* (LOC_Os08g32870), a major aroma-related gene [14,15,16], at position 20,379,793–20,386,061 on chromosome 8. 

An eight-base deletion and three single nucleotide polymorphisms (SNPs) in exon 7 of *OsBADH2* lead to a premature stop codon, resulting in a non-functional OsBADH2 and the production of aromatic rice [8,16,17]. Another study determined that a seven-base deletion in exon 2 also resulted in a non-functional OsBADH2 in aromatic rice [17]. The silencing of *OsBADH2* reportedly can convert non-aromatic rice to aromatic rice [18,19,20]. Moreover, mutations in the betaine aldehyde dehydrogenase gene *OsBADH1* (LOC_Os04g39020) at position 22,795,011–22,799,839 of chromosome 4 are also responsible for rice aroma [15,21]. 

A similar mechanism is present in aromatic soybean, in which a 2-base (TT) deletion in exon 10 of the betaine aldehyde dehydrogenase gene *GmBADH2* (Glyma05g01770) introduces a premature stop codon that ultimately results in a non-functional GmBADH2 [9]. Suppressing the expression of *GmBADH2* in a non-aromatic soybean variety via RNA interference is conducive to aroma formation [22]. The biosynthesis of 2AP in rice has been clarified. 

In the main 2AP biosynthesis pathway, polyamines (i.e., organic compounds with more than two amino groups) are converted to GAB-ald (i.e., immediate precursor of γ-aminobutyric acid; GABA), which is spontaneously converted to Δ1-pyrroline (i.e., the immediate precursor of 2AP and an important factor for regulating the 2AP biosynthesis rate) [23]. In non-aromatic rice, GAB-ald is converted to GABA by a functional BADH2 (encoded by *OsBADH2*), which ultimately inhibits 2AP biosynthesis. Conversely, GAB-ald cannot be converted to GABA by a non-functional BADH2 (encoded by *OsBADH2*), resulting in GAB-ald accumulation and 2AP formation in aromatic rice [24]. The molecular mechanism that regulates 2AP biosynthesis in soybeans might resemble that in rice.

Although multiple fragrance-related alleles have been identified in rice, only one type of sequence variation (TT deletion) has been identified among the aromatic soybean varieties. It remains unclear if there are other sequence variations, including in *GmBADH2*, that have the same effect on 2AP biosynthesis. 

In this study, new fragrant Chinese vegetable soybean cultivars were evaluated and characterized, and the candidate gene *GmBADH* sequences in aromatic and non-aromatic soybean varieties were compared. Additionally, the mechanisms underlying aroma production in the aromatic soybean cultivars Quxian No. 1 (QX1), ZK1754, and Xiangdou (XD) were analyzed. Quantitative real-time PCR (qRT-PCR) and CRISPR/Cas9 techniques were used to analyze the expression and characterize the function of *GmBADH2*. Furthermore, a functional marker was developed and validated in the F_2_ population.

## 2. Results

### 2.1. 2AP Content in Aromatic Soybean Cultivars

To examine the aroma profile in soybeans, we quantitatively analyzed the 2AP content in aromatic soybeans and non-aromatic soybeans using a gas chromatography and ion mobility spectrometry (GC-IMS) system. As expected, 2AP was detected in the aromatic varieties but not in the non-aromatic varieties. The average 2AP contents in QX1, XD, and ZK1754 were 6.33 ± 1.61, 6.04 ± 0.07, and 7.21 ± 0.51 μg/g, respectively. In contrast, 2AP was undetectable in the non-aromatic soybean cultivars Zhexian No. 8 (ZX8), Zhexian No. 9 (ZX9), and Danbohei (DBH).

### 2.2. Candidate Gene Sequence Acquisition and SNP/InDel Detection

In rice, *OsBADH1/2* are candidate genes for 2AP accumulation and aroma formation [25,26]. In an earlier study, a nucleotide deletion in *GmBADH2* significantly increased the seed 2AP level in the soybean cultivar Yuagari musume, resulting in an aromatic phenotype [22]. In this study, sequence information for *GmBADH1/2* was obtained by searching for the Williams 82 reference genome sequence in the Phytozome database (version 12.0). The results indicated that *GmBADH1* (Glyma06g19820) contains 15 exons and a coding sequence (CDS) comprising 1512 bp, whereas *GmBADH2* (Glyma05g01770) includes 15 exons and a CDS consisting of 1467 bp. These two homologous genes are 44.3% similar at the genome level, 65.4% similar at the transcript level, and 89.5% similar at the amino acid level.

**Figure 1:**
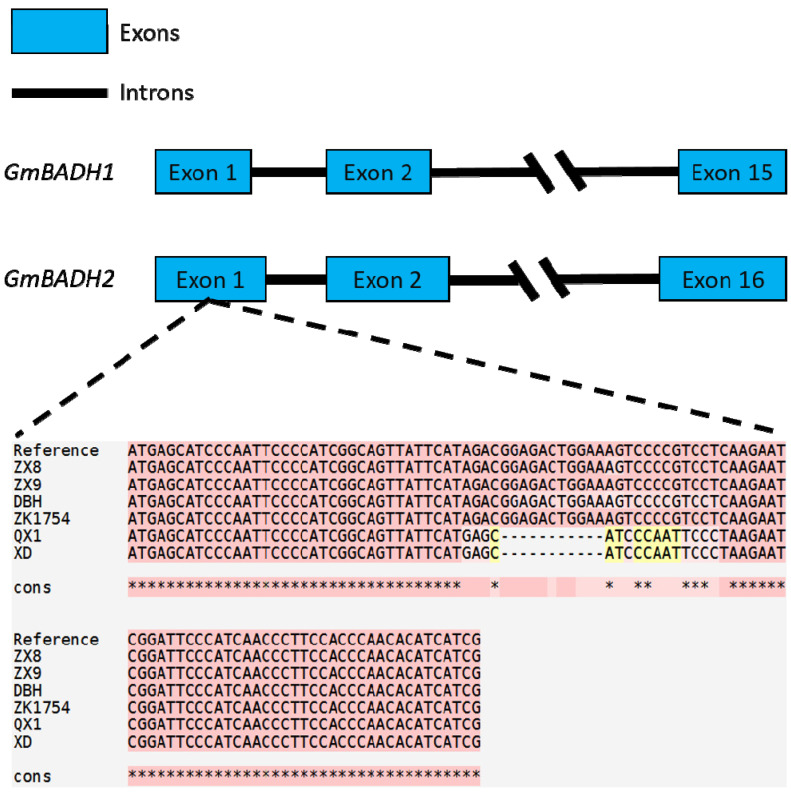
Alignment of part of the *GmBADH2* sequence (exon 1) in aromatic and non-aromatic soybean varieties. There were 10 SNPs and an 11-nucleotide deletion in exon 1 of *GmBADH2* in two aromatic soybean varieties. Aromatic soybean varieties: ZK1754, QX1, and XD; non-aromatic soybean varieties: ZX8, ZX9, and DBH; reference: Williams 82.

To detect potential mutation sites, full-length *GmBADH1/2* sequences from six soybean lines (DBH, XD, QX1, ZX9, ZX8, and ZK1754) were aligned with the corresponding sequences in the Williams 82 reference genome. The alignments revealed a lack of mutation in *GmBADH1*; however, a mutation with 10 SNPs and an 11-nucleotide (GGAGACTGGAA) deletion in the exon 1 region of *GmBADH2* were detected in the aromatic soybean cultivars XD and QX1 (Figure 1). The deletion of 11 nucleotides and the 10 SNPs in the aromatic varieties led to a frame shift and the introduction of premature stop codons and possibly a loss of function. Moreover, a 2-base (TT) deletion in the exon 10 region of *GmBADH2* in the aromatic soybean cultivar ZK1754 had similar effects, which were examined in an earlier study [22].

### 2.3. Genetic Analysis

As QX1 and XD were revealed to have the same sequence variation in *GmBADH2*, we selected QX1 and ZK1754 for an investigation of their inheritance patterns. An analysis of the 2AP contents of the DBH/QX1 and ZK1754/ZX8 F_1_ seeds (data not shown) indicated that the seeds had relatively low 2AP levels, similar to non-aromatic soybean DHB and ZX8 seeds. This suggested that the aromatic phenotype is under the control of allele(s) recessive in non-aromatic lines. 

In both F_2_ populations, the ratio of non-aromatic to aromatic individuals fit the 3:1 Mendelian model, indicating that the aromatic characteristic is inherited as a recessive allele of a single gene (Table 1). The subsequent examination of the F_2:3_ seeds derived from the DBH/QX1 and ZK1754/ZX8 crosses revealed the following three types of F_2_ plants: homozygous aromatic plants that resulted in F_2:3_ seeds with high 2AP contents, homozygous non-aromatic plants that resulted in F_2:3_ seeds with low 2AP contents, and heterozygous plants that resulted in F_2:3_ seeds with either high or low 2AP contents. The segregation of the aromatic, heterozygous, and non-aromatic plants fit a 1:2:1 ratio, which is in accordance with the single gene model (Table 1). The results indicated that the high 2AP contents in aromatic soybean cultivars QX1, XD, and ZK1754 were the result of a single gene.

### 2.4. GmBADH1 and GmBADH2 Expression Levels

**Figure 2:**
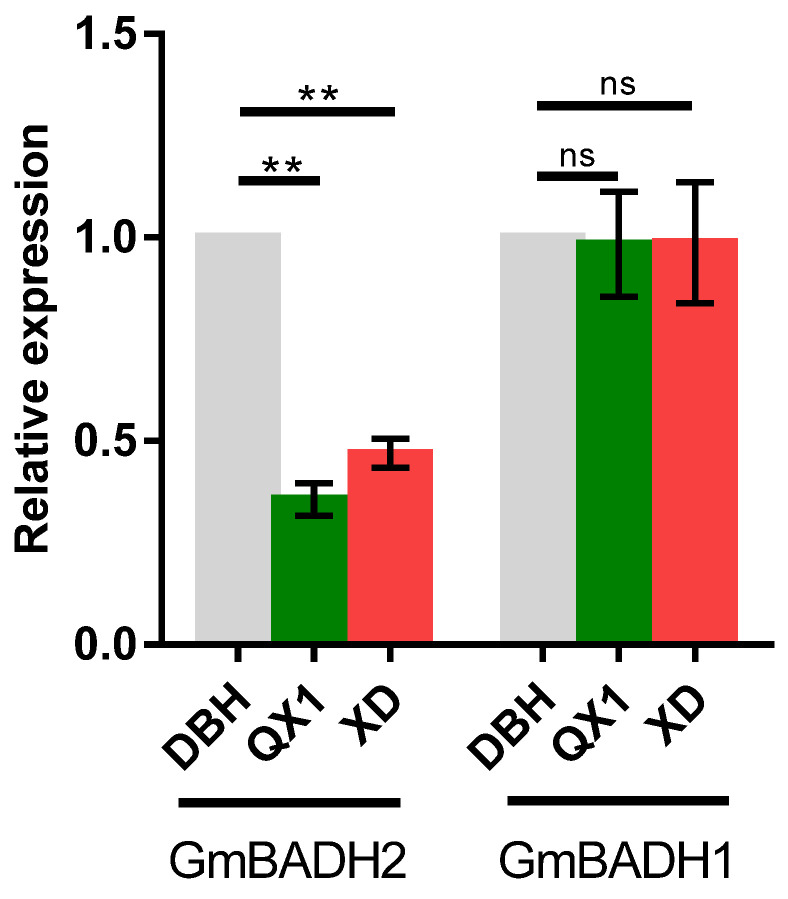
The *GmBADH1* (Glyma06g19820) and *GmBADH2* (Glyma05g01770) expression level in aromatic and non-aromatic soybean varieties. DBH: non-aromatic soybean variety. QX1 and XD: aromatic soybean varieties. Experiments were repeated using three independent biological replicates. Error bars: standard deviation. Significance was assessed by comparing the expression of each gene in the aromatic varieties with the corresponding expression in DBH; **, *p* < 0.01; ns, non-significant.

Due to the mutation in *GmBADH2* (Glyma05g01770) in the aromatic soybean varieties, we investigated the expression of this gene during the R6 seed stage as well as the expression of the homologous gene *GmBADH1* (Glyma06g19820). The results showed that the *GmBADH2* expression level in the R6 stage seeds was significantly lower in the aromatic soybean varieties (QX1 and XD) than in the non-aromatic soybean variety (DBH), whereas there were no significant differences in the *GmBADH1* expression levels (Figure 2). 

In this study, mutations in the aromatic varieties QX1 and XD led to a frame shift and the introduction of premature stop codons. Additionally, there was an inverse correlation between *GmBADH2* transcript abundance and the 2AP content, which was consistent with the results of a previous study [22] that determined that *GmAMADH2* (aminoaldehyde dehydrogenase) in soybean is expressed at much lower levels in aromatic varieties than in non-aromatic varieties.

### 2.5. Confirming the Function of GmBADH2 Using CRISPR/Cas9

**Figure 3:**
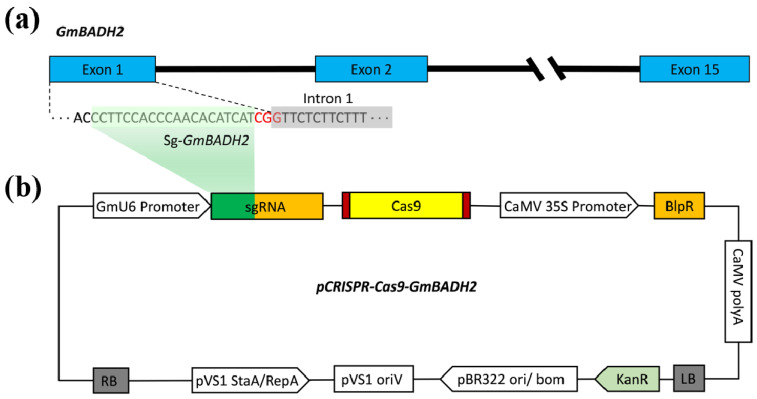
Targeted editing of *GmBADH2* using the CRISPR/Cas9 system. (**a**) Exon–intron structure of *GmBADH2*. Exons and introns are indicated by blue rectangles and a black horizontal line, respectively. The nucleotide sequence highlighted in green indicates the region targeted by the sgRNA designed in this study. The nucleotide sequence highlighted in gray indicates intron 1 and the red bases represent the PAM sequence. (**b**) Schematic diagram of the gene knockout vector pCRISPR-Cas9-GmBADH2; GmU6 indicates the soybean U6 promoter. Genes encoding Cas9 and BlpR were expressed under the control of the CaMV 35S promoter. BlpR, phosphinothricin resistance gene; LB, left border; and RB, right border.

**Figure 4:**
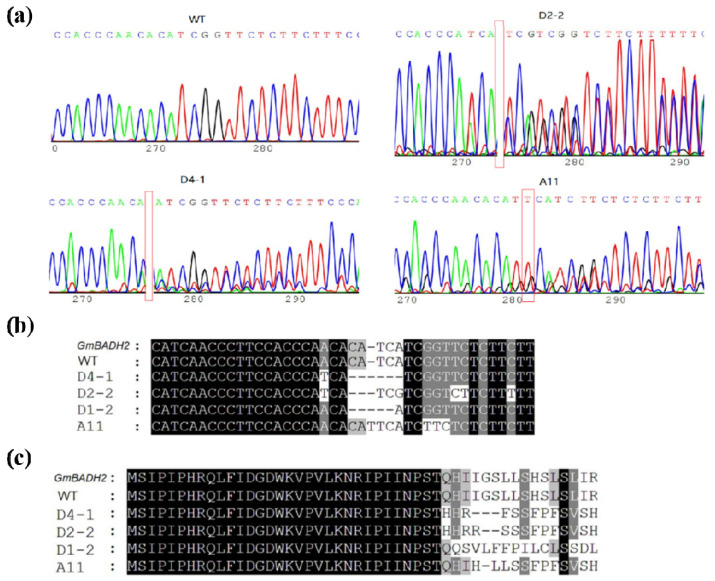
Mutations in T_0_ and T_1_ plants induced by sgRNA. (**a**) Sequencing peak plots of wild-type (WT) and T-DNA-positive T_0_ lines. Red boxes indicate possible mutation sites. (**b**) Alignment of the *GmBADH2* sequences of the T_1_ mutant and WT lines. WT, Tianlong No. 1; *GmBADH2*, Glyma05g01770. (**c**) Alignment of the GmBADH2 amino acid sequences of the T_1_ mutant and WT lines.

To confirm the function of *GmBADH2*, we designed a single-guide RNA (sgRNA) to induce a mutation in *GmBADH2*. Specifically, the sgRNA was designed to target *GmBADH2* exon 1 (target sequence: CCTTCCACCCAACACATCATCGG) (Figure 3a). After transforming wild-type (WT) soybean Tianlong No. 1 (TL1) with the pCRISPR-Cas9-GmBADH2 vector (Figure 3b) via *Agrobacterium tumefaciens*-mediated transformation, 30 positive plants were obtained in the T_0_ generation. The DNA extracted from leaf tissue was used to examine the CRISPR/Cas9-induced mutations in the target region on the basis of PCR and DNA sequencing analyses, and the results showed that nucleotide deletion occurred in T_0_ lines when compared with wild type TL1 (Figure 4a). We determined that three T-DNA-positive T_0_ lines had heterozygous-targeted mutations in the *GmBADH2* target region.

The seeds collected from the three self-pollinated T_0_ lines were sown under natural conditions (summer) in Hangzhou, China. Site-directed mutagenesis of *GmBADH2* was also detected in the target region in the T_1_ and T_2_ generations. Specifically, compared with the *GmBADH2* (Glyma05g01770) and WT (TL1) sequences, the sgRNA-induced mutations in D4-1, D2-2, and D1-2 were 5-, 2-, and 4-bp deletions, respectively (Figure 4b). All mutations that occurred in exon 1 were frame-shift mutations that altered the GmBADH2 amino acid sequence (Figure 4c).

A quantitative analysis of the 2AP contents in the T_2_ generation seeds using a GC-IMS system indicated that D1-2, D2-2, and D4-1 seeds contained significantly more 2AP than the WT control; however, there were differences in the 2AP contents among the mutants (Table 2).

### 2.6. Development of High-Throughput Molecular Markers for Aroma

**Figure 5:**
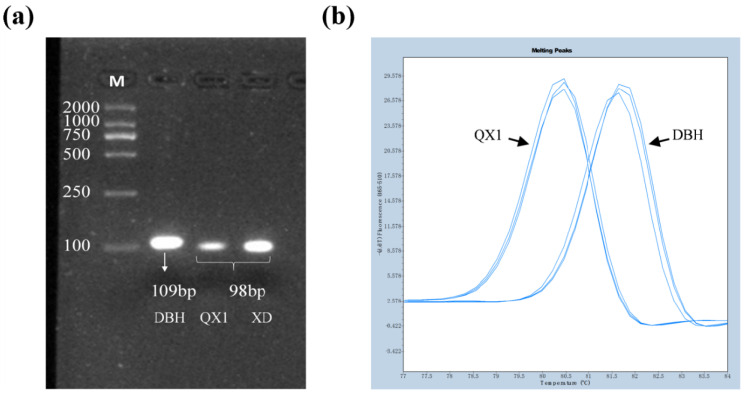
Development of molecular markers for the hybrid parents (DBH/QX1). (**a**) Agarose gel electrophoresis results indicated the amplified target sequences were 98 bp for the aromatic soybean varieties (QX1 and XD) and 109 bp for the non-aromatic soybean variety (DBH). (**b**) HRM peak profiles for DBH (82 °C) and QX1 (80 °C).

To develop molecular markers for the *GmBADH2* mutation site, we designed specific primer sequences. The sequence of interest was amplified by PCR to verify the specificity of the 05G-HRM primer pair. Although the PCR results confirmed that the primer pair specifically amplified the target sequence, the agarose gel electrophoresis analysis was unable to detect the 11-nucleotide deletion (Figure 5a). Next, we used this primer pair for a high-resolution melting (HRM) curve peak analysis, during which the melting curve peaks for *GmBADH2* were detected at 80 and 82 °C for the aromatic soybean (QX1) and the non-aromatic soybean (DBH), respectively (Figure 5b). Thus, this primer pair was appropriate for distinguishing between aromatic and non-aromatic soybean genotypes.

### 2.7. Validation of High-Throughput Molecular Markers

The genotyping results were validated by an analysis of 200 individual plants from the F_2_ generation derived from a cross between DBH and QX1. Additionally, the 2AP contents of the F_2:3_ seeds from 200 F_2_ individuals were determined. The HRM genotyping results indicated that 49 of the 200 F_2_ individuals had a melting temperature of 80 °C and homozygous recessive genotypes with an average 2AP content of 6.19 μg/g, which was similar to the 2AP content of the parent QX1 (Table 1). 

**Figure 6:**
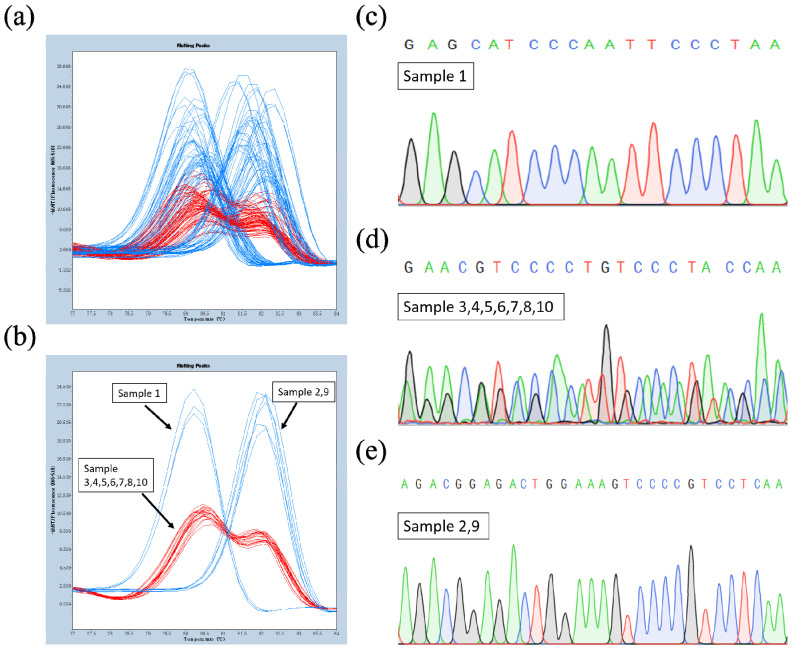
HRM curve peak plots and nucleotide sequences diagram of mutation site in F_2_ individuals. (**a**) HRM curve peak plots for the 200 individual plants from F_2_ hybrid population.(**b**) Ten individual strain HRM curve peak plots for the hybrid population. (**c**) Sequencing peak plot of the mutation homozygous (with 11-nucleotides) *GmBADH2* in the hybrid population. (**d**) Sequencing peak plot of the heterozygous genotypes of *GmBADH2* in the hybrid population. (**e**) Sequencing peak plot of the non-mutation homozygous (without 11-nucleotide deletion) *GmBADH2* in the hybrid population.

In contrast, 52 individuals had a melting temperature of 82 °C and homozygous dominant genotypes with undetectable amounts of 2AP, similar to the parent DBH, whereas 99 individuals had melting temperatures of 80 and 82 °C and heterozygous genotypes with an undetectable 2AP content (Table 1, Figure 6a). On the basis of the HRM results, 10 F_2_ individuals were validated by sequencing. 

The HRM typing results showed that sample 1 had melting temperatures of 80 °C, samples 2 and 9 had melting temperatures of 82 °C, and the other seven samples had melting temperatures of 80 and 82 °C (Table 3, Figure 6b).Accordingly, generated sequencing data indicated that an 11-nucleotide deletion (GGAGACTGGAA) was found in samples 1, 3, 4, 5, 6, 7, 8, and 10 (Figure 6c,d), and a longer sequence was found in samples 2 and 9 because of an undeleted 11-nucleotide sequence (Figure 6e). 2AP contents of 10 samples was also detected, and only sample 1 had a higher 2AP content (Table 3). The results reflect the co-segregation of mutation sites, aroma phenotypic traits, and HRM typing.

## 3. Discussion

Aroma is an important quality trait directly influencing the commercial value of soybean. The consumption of high-quality aromatic soybean and soybean products continues to increase. Hence, there is an urgent need for the production of new aromatic soybean varieties and the characterization of their aroma-related genes. We previously investigated the diversity of volatile compounds in vegetable soybean. Some cultivars (XD, ZK1754, and QX1) were revealed to have pleasant aromas and relatively high 2AP contents [27]. 

Notably, the 2AP contents in these cultivars were higher than the corresponding contents determined in earlier studies [9,22,28] possibly because of the differences in the examined tissues and in the extraction and detection methods used among studies. Previous research identified 2AP as the key flavor compound in the aromatic vegetable soybean known as *dadacha-mame* [3] and revealed that 2AP levels are higher in aromatic soybeans than in non-aromatic soybeans [9,22]. We detected high 2AP contents in aromatic soybean seeds, confirming the contribution of this volatile compound to the soybean aroma profile. The aroma profiles of other species have also been thoroughly investigated [5,6,28,29,30].

Although some Chinese soybean cultivars have an aromatic phenotype, the underlying genetic mechanism remains unknown. However, *GmAMADH2*, which encodes an aminoaldehyde dehydrogenase, is reportedly responsible for the fragrant phenotype of aromatic vegetable soybean from Japan [22]. The sequence of *GmAMADH2*, which regulates aroma biosynthesis in soybeans, is highly similar to the sequence of a gene encoding betaine aldehyde dehydrogenase (BADH). Although the complete 2AP biosynthesis pathway in plants has not been fully elucidated, a key 2AP biosynthesis pathway regulated by BADH2 was proposed for various plant species, including rice [12], sorghum [31], winter melon [6], and soybean [9]. 

Additionally, BADH2 belongs to the ALDH10 family, which includes NAD(P)^+^-dependent enzymes [32,33] and catalyzes the conversion of betaine aldehyde to betaine [34]. A non-functional BADH2 results in the formation of l-pyrroline from GAB-ald, instead of GABA, which leads to an increase in 2AP production and aroma formation. The current study involved a genetic analysis of three Chinese aromatic vegetable soybean cultivars. The *BADH2* sequences in three aromatic cultivars (XD, ZK1754, and QX1) and three non-aromatic cultivars (ZX8, ZX9, and DBH) were examined. As expected, *GmBADH2* was identified as the candidate mutated gene contributing to the aromatic phenotype of soybean seeds. 

A loss-of-function mutation in this gene will lead to the accumulation of the aroma compound 2AP, thereby, enhancing the fragrance of soybean seeds. The absence of a detectable mutation was consistent with the lack of aroma in DBH, ZX8, and ZX9. In contrast, a mutated *BADH2* in XD, ZK1754, and QX1 was revealed to be associated with the production of the aroma compound 2AP in these varieties. Interestingly, the mutation sites identified in this study differ from those detected in earlier studies [9,22], which reflects the varietal differences in aroma production. However, both mutations lead to decreased *GmBADH2* expression levels [22]. 

Previous research indicated that a 2-bp deletion in exon 10 of *GmAMADH2* was responsible for soybean aroma formation [22]. In the current study, this 2-bp deletion was detected in ZK1754, which may explain the observed changes in the 2AP content of this variety. In XD and QX1, we detected an 11-nucleotide deletion and 10 SNPs, which resulted in a frame shift and the introduction of internal stop codons that likely represent loss-of-function mutations. 

In rice, different types of *OsBADH2* mutations may be the cause of the reported variations in 2AP accumulation [35,36]; however, there has been relatively little research regarding the diversity in the 2AP accumulation among soybean mutants with a mutated *GmBADH2*. The identification of new *BADH2* mutations in different genetic backgrounds and different alleles may provide researchers and breeders with important genetic information relevant for future studies aimed at enhancing soybean seed aromas and the breeding of new soybean varieties.

The CRISPR/Cas9 gene-editing system is useful for verifying the function of *GmBADH2* and for generating new mutants. In this study, we used CRISPR/Cas9 technology to introduce new mutations in exon 1 of *GmBADH2* as previously described in rice [37,38,39]. A comparison between the new soybean mutants and their WT parent indicated that the mutants had a higher 2AP content and an aromatic phenotype, although the 2AP content differed significantly among the mutants. These new aromatic soybean mutants were examined to preliminarily confirm the function of *GmBADH2*. Furthermore, they may be useful in soybean breeding programs.

On the basis of published research, 2AP is considered to be the volatile compound responsible for the aromas of various cereal products as well as vegetable and animal food products [40]. It has a critical effect on the economic value of food products because it can influence consumer preferences. The aroma trait is one of the most challenging traits for plant breeders to select for because of the difficulties associated with evaluating the aroma phenotype [8]. As the soybean aroma is very strong and easily detected by the human nose, many breeding programs focused on improving soybean aroma have used subjective sensory methods [16,41]. These methods are also applicable for assessing soybean flavors [41]. 

However, sensory methods can only qualitatively characterize the aroma profile (i.e., the presence or absence of aroma). They are generally inappropriate for evaluating aroma intensity, which may vary among assessors. Several studies have used an HS-GC-IMS system to analyze the volatile compounds of wine, eggs, jujube fruits, and honey [42,43,44,45]. These methods have many advantages over other methods, including speed of detection, high sensitivity, and simple sample preparation steps. In addition to the HS-GC-IMS detection method used in this study, 2AP contents can also be quantified by GC-MS [6,18,35,46,47,48]. 

However, there are also many limitations to all of these methods (e.g., the inability to simultaneously process many samples, low efficiency, laborious production of substantial amounts of tissue samples, and time-consuming analyses). Increasing the efficiency, cost-effectiveness, and throughput of genotyping methods is a major challenge for breeding programs. A co-dominant PCR-based marker for the aroma trait in soybeans has been designed on the basis of the 2-bp deletion in *GmAMADH2* [7]. This marker may be exploited by aromatic soybean breeding programs relatively easily. In the current study, we developed high-throughput molecular markers for genotyping. These markers may enable researchers to rapidly screen for aroma-related genes that cannot be easily detected using other methods. 

The aroma-related genotype with the 11-nucleotide deletion and 10 SNPs was associated with two aromatic soybean varieties as well as the aromatic progeny of the F_2_ population with seeds containing 2AP. Moreover, the non-aromatic genotype was associated with two non-aromatic varieties and the non-aromatic progeny. The perfect co-segregation of the marker genotypes and aroma phenotypes confirmed *GmBADH2* is the major gene conferring the aroma. Furthermore, the markers may be useful for the efficient molecular breeding of soybean with enhanced aroma traits.

## 4. Materials and Methods

### 4.1. Plant Materials

In this study, the aromatic soybean varieties were QX1, ZK1754, and XD, whereas the non-aromatic soybean varieties were ZX8, ZX9, and DBH. Additionally, QX1 was created by the Quzhou Academy of Agricultural and Forestry Sciences and released by the Zhejiang government. Both ZX8 and ZX9 were bred by the Nuclear Institute of Zhejiang Academy of Agricultural Sciences and released by the Zhejiang government. Finally, XD, ZK1754, and DBH are soybean landraces.

Two F_2_ populations from the QX1/DBH and ZK1754/ZX8 hybridizations were developed to assess the relationship between base mutations and the aromatic phenotype. Both populations comprised 200 individuals, each homozygous for the aromatic or non-aromatic trait. The F_2_ individuals were derived from five individual F_1_ plants and classified after analyzing the 2AP contents of F_2:3_ fresh seeds as described in the following section. The 400 individuals in the two F_2_ populations and all selected cultivars were grown on the experimental farm of Zhejiang Academy of Agricultural Sciences in Hangzhou, Zhejiang province in 2020.

### 4.2. Quantitative Analysis of the 2AP Content

The 2AP contents of all cultivars were measured by HS-GC-IMS. More specifically, 50 fresh pods were harvested at the R6 stage and then boiled in water for 5–10 min. The boiled seeds were crushed and then placed in an incubator for the HS-GC-IMS analysis. The GC-IMS conditions were as follows: analysis time, 20 min; column type, FS-SE-54-CB-1, 15 m with an internal diameter of 0.53 mm; column temperature, 60 °C; drift gas flow, 150 mL/min; carrier gas/drift gas, nitrogen; IMS temperature, 45 °C; injection volume, 400 μL; incubation time, 15 min; incubation temperature, 60 °C; injection temperature, 65 °C; and incubation speed, 500 rpm. The 2AP concentration was determined using an external standard.

Three independent fresh pod samples of each soybean line/individual were used to determine the 2AP content. Each soybean pod sample was analyzed three times, and then the mean value was calculated. Data were expressed as the mean ± standard deviation and analyzed by a one-way analysis of variance (ANOVA) followed by the Duncan test.

### 4.3. Genetic Analysis and Gene Identification

Aromatic soybean cultivars QX1 and ZK1754 were crossed with non-aromatic varieties DBH and ZX8, respectively, after which 200 F_2_ seeds were harvested from individual F_1_ plants. Segregating F_2_ seeds for the aromatic phenotype were used for the subsequent analysis. Seeds from individual F_2_ plants (F_2:3_ seeds) were harvested and then 11–23 seeds per F_2_ plant were analyzed using the HS-GC-IMS method to determine their 2AP contents. The trait and gene segregation ratio analyses were verified by the Chi-squared test.

As a previous study revealed the 2AP content is a major factor influencing plant aromas, we speculated that *GmBADH2*, which encodes a betaine aldehyde dehydrogenase that catalyzes the key step of the 2AP biosynthesis pathway, may contribute to the soybean aroma phenotype [46]. Hence, we searched for soybean (*G. max*) betaine aldehyde dehydrogenase genes in the NCBI (http://www.ncbi.clm.nih.gov/sites/entrez (accessed on 20 October2021)) and Phytozome (http://www.phytozome.net/search.php?show=blast&method=org_Gmax (accessed on 20 October 2021)) databases using the *GmBADH1/2* cDNA sequences as queries.

### 4.4. DNA Extraction and Gene Sequencing

The total DNA was extracted from the young tender leaves of 400 individuals in the F_2_ populations resulting from the QX1/DBH and ZK1754/ZX8 hybridizations and the six selected cultivars according to the CTAB method [49]. The quality of the extracted DNA samples was assessed by 1.2% agarose gel electrophoresis. Using the NanoDrop spectrophotometer (Thermo, Waltham, MA, USA), all DNA sample concentrations were adjusted to 50 ng/μL for the subsequent PCR amplifications.

Fourteen primer pairs (Table 4) were designed to amplify the full-length *GmBADH1* (Glyma06g19820) and *GmBADH2* (Glyma05g01770) genomic DNA and cDNA sequences, with 100- to 200-bp overlapping sequences between adjacent fragments. The primers were designed using the Primer Premier 5 software and the soybean Williams 82 reference genome sequence (http://www.phytozome.net/cgi-bin/gbrowse/soybean (accessed on 20 October 2021)). All primers were synthesized by Tsingke Biotechnology Co., Ltd. (Beijing, China). The PCR-amplified products were used for genomic DNA and cDNA sequencing. 

The PCR amplification was performed as previously described [50]. The amplified products were separated by 1.2% agarose gel electrophoresis, excised from the gel, and purified using the AxyPrep DNA Gel Extraction Kit (Vitagen, Hangzhou, China). Genomic DNA and cDNA fragments were directly sequenced by Tsingke Biotechnology Co., Ltd. (Beijing, China). Forward and reverse sequences were assembled into a contig using SnapGene (version 4.1.9) and aligned using Bio-Edit (version 8.1.0).

### 4.5. RNA Isolation and qRT-PCR Analysis

The total RNA was extracted from soybean pods harvested at the R6 stage using the Polysaccharide and Polyphenol Total RNA Isolation kit (Bioteke, Beijing, China). Contaminating genomic DNA was eliminated using RQ1 RNase-Free DNase (Promega, Madison, WI, USA). The concentrations of all RNA samples were quantified using the NanoDrop spectrophotometer and adjusted to approximately 1000 ng/μL for the qRT-PCR analysis. The quality of the RNA samples was evaluated by 1.2% agarose gel electrophoresis.

First-strand cDNA was synthesized using Oligo-dT (50 μM) as the primer and the Biomarker Script II Enzyme Mix (10×) (Biomarker, Beijing, China) according to the manufacturer’s instructions. A single bulk cDNA synthesis reaction was performed, and the cDNA was diluted to enable the completion of many PCR amplifications and to minimize the potential differences between cDNA synthesis reactions.

The expression of soybean *GmBADH1* was monitored using the following gene-specific primers: forward 5′-TACATATCTGATGAACCGTGGG-3′; reverse 5′-GATAAAATCAACTCAGCCCGTC-3′. These primers were designed to amplify a 200-bp cDNA fragment. Additionally, the following gene-specific primers were used to monitor the expression of soybean *GmBADH2*: forward 5′-TGAAGCGGGTGCTCCTTTAG-3′ and reverse 5′-AATATGGTCCATTCAGCAGC-3′. These primers were designed to amplify a 195-bp cDNA fragment. 

Expression levels were normalized against the expression of a soybean *Actin* encoding gene (Glyma.02G091900) (forward primer 5′-GCATTGCAGATTGTAACCCTTT-3′; reverse primer 5′-CTCTCCTCACCTTCTGCAAATA-3′; amplified product size of 96 bp). The qRT-PCR analysis was performed using the SYBR^®^ Green Real-time PCR Master Mix Plus (TOYOBO Biotech Co., Ltd., Shanghai, China) and the LightCycler^®^ 480 instrument (Roche, Basel, Switzerland). The reactions were conducted in triplicate for each biological replicate. 

The 20-μL reaction mixtures comprised 2 μL template, 10 μL SYBR Green Real-time PCR Master Mix Plus, 1.2 μL each primer (10 μM), 2 μL Plus Solution, and 3.6 μL ddH_2_O. The PCR conditions were as follows: Program 1: one cycle of 95 °C for 30 s and a ramp rate of 20 °C/s. Program 2: analysis mode: quantification, 50 cycles of 95 °C for 5 s, 55 °C for 10 s, and 72 °C for 15 s, and a ramp rate of 20 °C/s; acquisition mode: 72 °C once. Program 3: analysis mode: melting curves, one cycle of 95 °C for 10 s, 70 °C for 10 s, 95 °C for 0 s, and 40 °C for 10 s; acquisition mode: 95 °C continuous. The relative gene expression levels were quantified according to the 2^−ΔΔCt^ method.

### 4.6. pCRISPR-Cas9-GmBADH2 Vector Construction

The targets were designed, and off-targets were detected using the Biogle Genome Editing Center online tool (http://biogle.cn/index/excrispr (accessed on 20 October 2021)). Pairs of DNA oligonucleotides for the two sgRNAs were synthesized by Tsingke Biological Technology (Beijing, China) and annealed to generate dimers, which were subsequently integrated into the BGK41 vector (Biogle, Hangzhou, China) as previously described [51]. The sgRNA and *Cas9* sequences were expressed under the control of the U6 and CaMV 35S promoters, respectively. Recombinant plasmids were introduced into *A. tumefaciens* strain EHA101 cells for the following soybean transformation.

### 4.7. Soybean Transformation and Screening for Mutations

Soybean cultivar Tianlong No. 1 was used for tissue cultures and transformation as previously described [52]. Transgenic plants were identified by a PCR amplification using *Cas9*-specific primers and sequencing. Heterozygous mutations were detected as superimposed peaks from the target sites to the end of the sequence, whereas the WT sequence and homozygous mutations had no superimposed peaks at the target sites.

### 4.8. Development and Validation of Aroma-Related Molecular Markers

Target sequences were amplified for an HRM analysis using the 05G-HRM primer pair (forward primer 5′-TCTCTCCTTTGACGACAAGATGAGC-3′; reverse primer 5′-CGGGTTGATGATGGGAATCCGAT-3′; amplified product size of 109/98 bp) and genomic DNA extracted from the samples to be tested. The LightCycler^®^ 480 II instrument and the LightCycler^®^ 480 High Resolution Melting Master kit were used for HRM genotyping. Reaction mixtures comprised 2 μL genomic DNA, 10 μL 2× LightCycler^®^ 480 High Resolution Melting Master Mix, 0.2 μL each upstream and downstream primer (10 μM), 2 μL MgCl_2_, and sterile water for a final volume of 20 µL. The reaction conditions were as follows: 95 °C for 10 min; 45 cycles of 95 °C for 10 s, 57 °C for 15 s, and 72 °C for 20 s; and 95 °C for 1 min, 40 °C for 1 min, and 65 to 95 °C for 10 s. After the reaction was completed, the amplification and melting curves were analyzed to ensure that they were acceptable.

## 5. Conclusions

In this study, we detected 10 SNPs and an 11-nucleotide deletion in the *GmBADH2* gene of the aromatic vegetable soybean varieties QX1 and XD as well as a 2-bp deletion in the *GmBADH2* gene of the aromatic vegetable soybean variety ZK1754, which resulted in a significant decrease in the *GmBADH2* expression level relative to the corresponding expression level in non-aromatic soybeans. Additionally, *GmBADH2* was functionally characterized using a CRISPR/Cas9 system. These results suggest that *GmBADH2* is a key gene for the 2AP production in three new aromatic soybean cultivars. 

Furthermore, HRM markers were developed on the basis of the *GmBADH2* mutations. The molecular markers and aromatic phenotypes co-segregated in 200 individuals in the QX1/DBH F_2_ population, indicating the aromatic trait weremainly the result of a mutated *GmBADH2*. Thus, the results of this study may be relevant for future comprehensive analyses of the molecular mechanism underlying 2-AP accumulation in soybean seeds as well as the marker-assisted breeding of aromatic vegetable soybean cultivars for commercial production.

## Figures and Tables

**Table 1:** Genetic analysis and phenotypes of the aroma trait in F_2_ progeny.

Crosses	QV1*DBH		ZK1754*ZX8		QV1*DBH	QV1*DBH
	Number				Melting Peak Temperature	2AP Mean Content (μg/g)
	Observed	Expected	Observed	Expected		
Genotype (expected ratio is 1:2:1)						
AA	52	50	45	50	82 °C	-
Aa	99	100	108	100	80 and 82 °C	-
aa	49	50	47	50	80 °C	6.19 ± 1.24
Total	200	200	200	200		
χ^2^-value	χ^2^_(1:2:1)_ = 0.11		χ^2^_(1:2:1)_ = 0.72			
Phenotype (expected ratio is 3:1)						
Non-aroma	151	150	153	150	82 or 80 and 82 °C	-
Aromatic	49	50	47	50	80 °C	6.19 ± 1.24
Total	200	200	200	200		
χ^2^-value	χ^2^_(3:1)_ = 0.027		χ^2^_(3:1)_ = 0.24			

χ^2^0.05,1 = 3.84 for 3:1 and χ^2^0.05,2 = 5.99 for 1:2:1, *p* = 0.05; “-” indicates 2AP was undetectable (GC-IMS analysis).

**Table 2:** GC-IMS quantification of 2AP contents in the seeds of T_2_ mutants with CRISPR/Cas9-induced mutations.

Samples	2AP (μg/g)		
	Mean	SD	RSD
D1-2	4.16 **	1.12	1.32
D2-2	7.72 **	1.54	1.65
D4-1	5.17 **	1.70	1.97
WT	0.81	0.31	0.47

SD: standard deviation; RSD: relative standard deviation; WT: wild-type (TL1); ** *p* < 0.01.

**Table 3:** 2AP content of 10 F_2_ individuals.

Samples	2AP (μg/g)		
	Mean	SD	RSD
1	6.17 **	1.31	1.19
3, 4, 5, 6, 7, 8, 10	-	-	-
2, 9	-	-	-

SD: standard deviation; RSD: relative standard deviation; ** *p* < 0.01; “-” indicates 2AP was undetectable.

**Table 4:** Primers designed for the candidate genes *GmBADH1* (Glyma06g19820) and *GmBADH2* (Glyma05g01770).

Primer	Gene Name	Product Size (bp)	Sequence (5′-3′)
06G-EX1-2	*GmBADH1*	600	**F:** AAAAGATCTGTGATGACTCATTAGCAAG **R:** GAACCTTTGAAGCGATGGC
06G-EX3		759	**F:** CAAAGGCAAAGATTGGTCTTCAG **R:** GTTAAATGTCACCACTCACCAGG
06G-EX4-5		785	**F:** GGAAAGCTTGAAGCAATTGATTG **R:** ACTTCTCTGCATATTTCAGCCAG
06G-EX6-8		719	**F:** TCTGAATTGGCATCTGTGTATGTTC **R:** GGTTGTCCATAACAAAATAAAAGGCAC
06G-EX9-10		777	**F:** GTTTTTGAGGATGTTGACCTTGATAAGAG **R:** ACTGATTGCCAGACAAAGTGTTTAAC
06G-EX11-12		788	**F:** CTCTTTCTGATTAATGTACTTCTGCC **R:** GGGGATAAAAAATAACGATGACTCAAA
06G-EX13-14		741	**F:** GCTATTGAACTAGCAAATGACACAC **R:** ATTTTCCACAAATAACTATTAGATGAGGG
06G-EX15		789	**F:** GAATCTTATTTCCTATATCAGTGAATGAGG **R:** TTAACTAGAAAACACAACAAAAACATTTAAGAAATC
05G-EX1-3	*GmBADH2*	686	**F:** TTTAGGATAAAGAAGGAGAGACTGGACTAGC **R:** GGTCAGCATAGAACTCAAAGCAACC
05G-EX4-6		786	**F:** GCTCGATGAAGCCGCCTG **R:** ACCTTGTGTGCAACAATGTGAGAC
05G-EX7-9		842	**F:** CCAAAATTGCGGCCACGC **R:** CCAAGGGATCAGAAATTTTGATGTTTTTGACC
05G-EX10		1311	**F:** GCTGAATGGACCATATTTGGTTGC **R:** GAGAGCAATTAATCCACACAATTCCAGC
05G-EX11-13		777	**F:** GAGAAGGAGTCAGAGAAGGAACCTTC **R:** GAGAGCAATTAATCCACACAATTCCAGC
05G-EX14-15		1036	**F:** GTGAGCGCATTACTAAGGTGAGAC **R:** CAAACATTAGAAGCATGTGGTGGAGC

## Data Availability

The datasets generated and/or analyzed during the current study are available from the corresponding author on reasonable request.
